# Subclavian Steal Syndrome as the Initial Presentation of Takayasu’s Vasculitis in a Young Caucasian Female

**DOI:** 10.7759/cureus.37940

**Published:** 2023-04-21

**Authors:** Beatrice E Torere, Henry O Aiwuyo, Max Rash, Gene Gerlach, Noah Russell, Amy Robinson Dolye

**Affiliations:** 1 Internal Medicine, North Mississippi Medical Center, Tupelo, USA; 2 Internal Medicine, Brookdale University Hospital Medical Center, Brooklyn, USA

**Keywords:** immune-mediated, caucasian female, vasculitis, takayasu, subclavian steal syndrome

## Abstract

Takayasu arteritis (TAK) is a rare but well-known inflammatory disease affecting large vessels that leads to thickening, narrowing, occlusion, or dilation of the affected arteries. The overall effect of the disease is arterial insufficiency of the brain and/or the distal part of the affected vessel. Subclavian steal syndrome has been observed as a form of presentation where there is occlusion of the proximal subclavian artery that results in a reversal of flow in the ipsilateral vertebral artery, consequently diverting or ‘stealing’ blood from the contralateral vertebral artery. Our patient is a 34-year-old Caucasian female presenting with subclavian steal syndrome as the initial presentation of TAK. She presented to the emergency department following a syncopal episode and six months prior history of intermittent lightheadedness, vertigo, left upper extremity pain, numbness, and tingling which was said to be aggravated with activity and alleviated with rest. Examination findings revealed non-palpable left brachial and radial pulses of the upper limb with an inaudible blood pressure reading on the ipsilateral side and blood pressure of 113/70 mmHg on the contralateral arm.

Investigation revealed elevated acute phase reactant, normocytic anemia, and inflammation of the aorta on imaging. She was evaluated by the vascular surgery team who recommended medical management. The patient was managed with steroids and methotrexate, and her symptoms improved significantly with the normalization of laboratory findings. She is currently being followed up by the vascular surgery and rheumatology teams. We emphasize the importance of understanding the varied clinical spectrum of TAK and the need to have a high index of suspicion for TAK in a young female with recurrent syncope and unilateral upper extremity intermittent numbness and paresthesia.

## Introduction

Takayasu arteritis (TAK) is an uncommon, chronic, immune-mediated, granulomatous inflammation of large vessels that leads to thickening, narrowing, occlusion, or dilation of affected arteries [1,2]. It commonly affects the aorta and its branches (left common carotid, brachiocephalic, and left subclavian), leading to vascular narrowing, occlusion, and weakened peripheral pulses [1]. Hence, TAK is occasionally called “pulseless disease” [1]. The exact etiology of TAK is unknown [3]. TAK can cause occlusion of the subclavian artery, leading to vertebrobasilar ischemia that presents as subclavian steal syndrome [4].

Women are affected in greater than 80% of cases, with the age of onset at less than 40 years [5]. TAK has a global incidence, with the highest prevalence found in Asian women [5].

Subclavian steal syndrome is the occlusion of the proximal subclavian artery, which leads to flow reversal in the ipsilateral vertebral artery and the “stealing” of blood from the contralateral vertebral artery [4]. Subsequently, this causes arterial insufficiency of the brain and upper extremity supplied by the occluded vessel. Clinical features of subclavian steal syndrome include lightheadedness, syncope, orthostasis, headaches, strokes, and convulsions [6].

The American College of Rheumatology developed a classification criterion to help diagnose TAK [7]. Patients are said to have TAK if at least three of the six criteria are present: age at disease onset ≤40 years, claudication of the extremities, decreased pulsation of one or both brachial arteries, a difference of at least 10 mmHg in systolic blood pressure between the arms, bruit over one or both subclavian arteries or the abdominal aorta, and arteriographic narrowing or occlusion of the entire aorta, its primary branches, or large arteries in the proximal upper or lower extremities that is not due to arteriosclerosis, fibromuscular dysplasia, or other causes [7].

The clinical presentation of TAK is divided into two phases: a systemic phase and an occlusion phase [8]. The systemic phase is the active inflammation phase [1,8]. The patient can present with various symptoms such as fever, night sweats, arthritis, or myalgias [1,8,9]. These non-specific symptoms often lead to delays in diagnosis [8]. The systemic phase is followed by the occlusive phase, during which patients develop symptoms caused by arterial narrowing and occlusion [9]. The systemic phase is the most common initial presentation of TAK [8]. However, the occlusive phase may present first, or they could simultaneously have features of both phases [1,8].

In this report, we present a case of a 34-year-old Caucasian female presenting with subclavian steal syndrome as the initial presentation of TAK.

## Case presentation

The patient is a 34-year-old Caucasian female who presented to the emergency department following a syncopal episode. She reported six months history of intermittent lightheadedness, vertigo, left upper extremity pain, numbness, and tingling. Symptoms were aggravated by physical exertion and improved with rest. Three months before the presentation, she developed frequent lightheadedness, progressive left upper extremity paresthesia, extreme fatigue, cramping, pain in her left arm with use, recurrent syncopal episodes, transient ischemic attack, and daily intermittent numbness to the left side of her face.

Physical examination revealed non-palpable left brachial and radial pulses of the left upper limb, blood pressure of the right upper limb of 113/70 mmHg, and inaudible in the left upper limb.

Laboratory tests showed normocytic anemia, an erythrocyte sedimentation rate (ESR) of 66 mm/hour, and a C-reactive protein (CRP) of 1.5 (Table 1).

**Table 1:** Remarkable laboratory findings MCV = mean corpuscular volume

Test	Findings	Reference range
White blood cell count (x1,000/UL)	6.9	5.0-10.0
Hemoglobin (mg/dl)	11.8	12.0-16.0
MCV (FL)	90.7	82-96
Platelet count (x1,000/UL)	285	150-400
Erythrocyte sedimentation rate (mm/hr.)	66	0-20
C-reactive protein (mg/dl)	1.5	<1.0
Rapid plasma reagin, qualitative	Non-reactive	
Serum creatinine (mg/dl)	1.2	0.6-1.0
Troponin (ng/ml	<0.012	0.000-0.120
Fluorescent Antinuclear Antibody (FANA) evaluation	Negative	
Blood culture	No growth	

**Figure 1:**
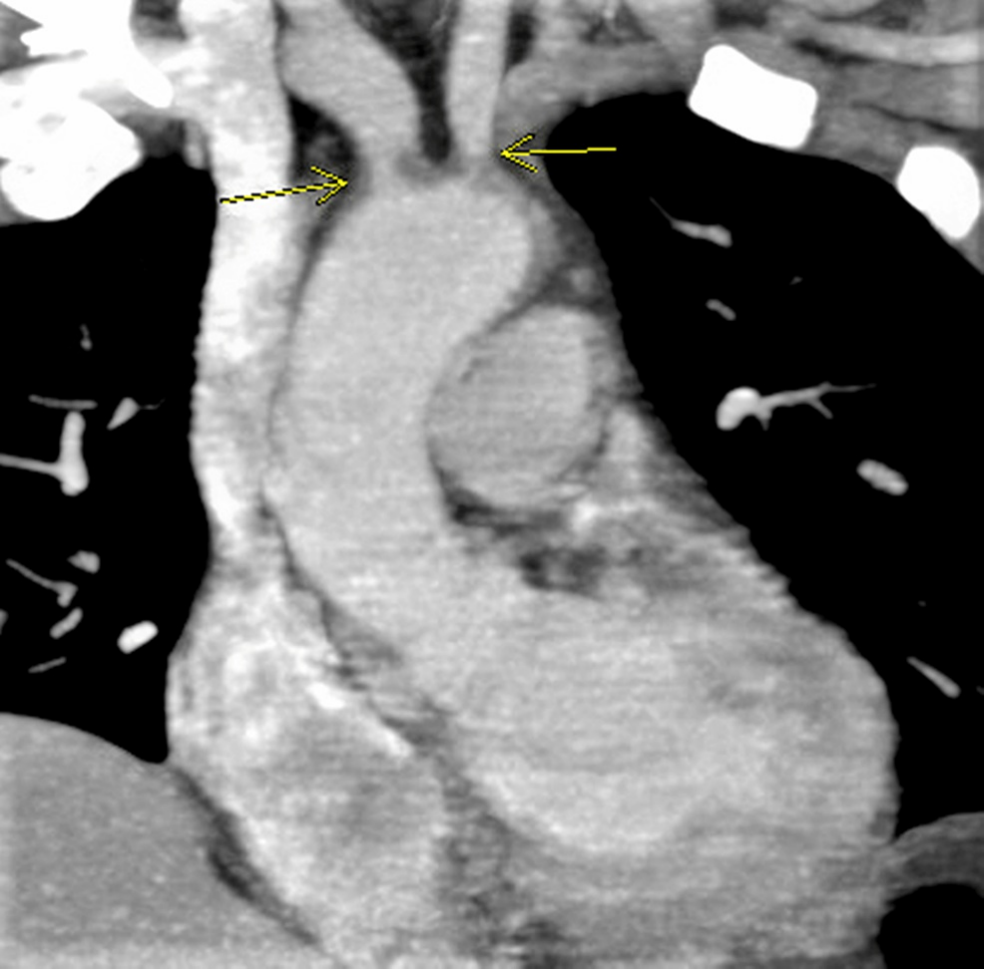
Computed tomography angiography of the neck

Computed tomography angiography of the neck (CTA neck) showed prominent mural thickening involving the transverse thoracic aorta extending to the mid-descending segment consistent with aortitis; concentric mural thickening in the proximal left common carotid artery at its origin produced a greater than 90% diameter stenosis in this location. Chronic occlusion was seen at the origin of the left subclavian artery. This vessel reconstitutes at the level of the left vertebral artery. The remainder of the carotid vasculature is widely patent throughout its course. Bilateral vertebral arteries are codominant and widely patent: 70% innominate artery stenosis, 70% left common carotid artery stenosis, and occluded left subclavian artery (Figure 1).

**Figure 2:**
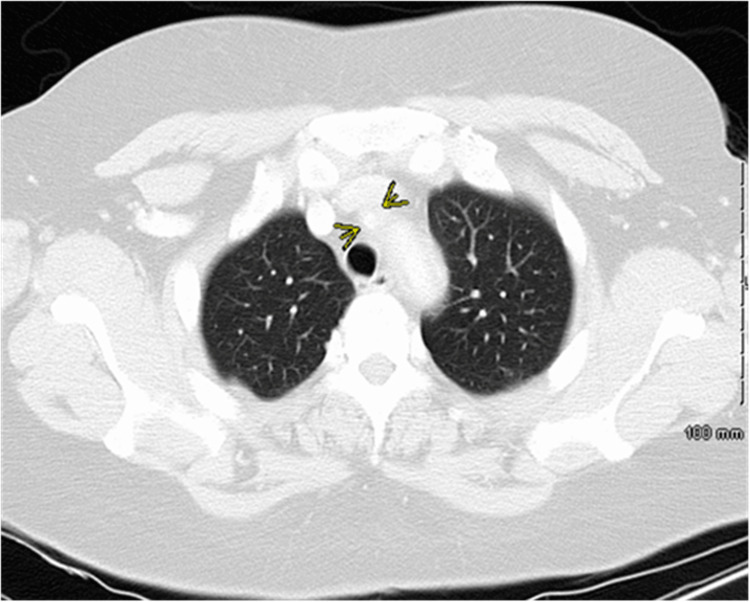
Computed tomography angiography of chest, axial view The yellow arrow indicates mural thickening that extends into the proximal aortic arch branching vessels.

**Figure 3:**
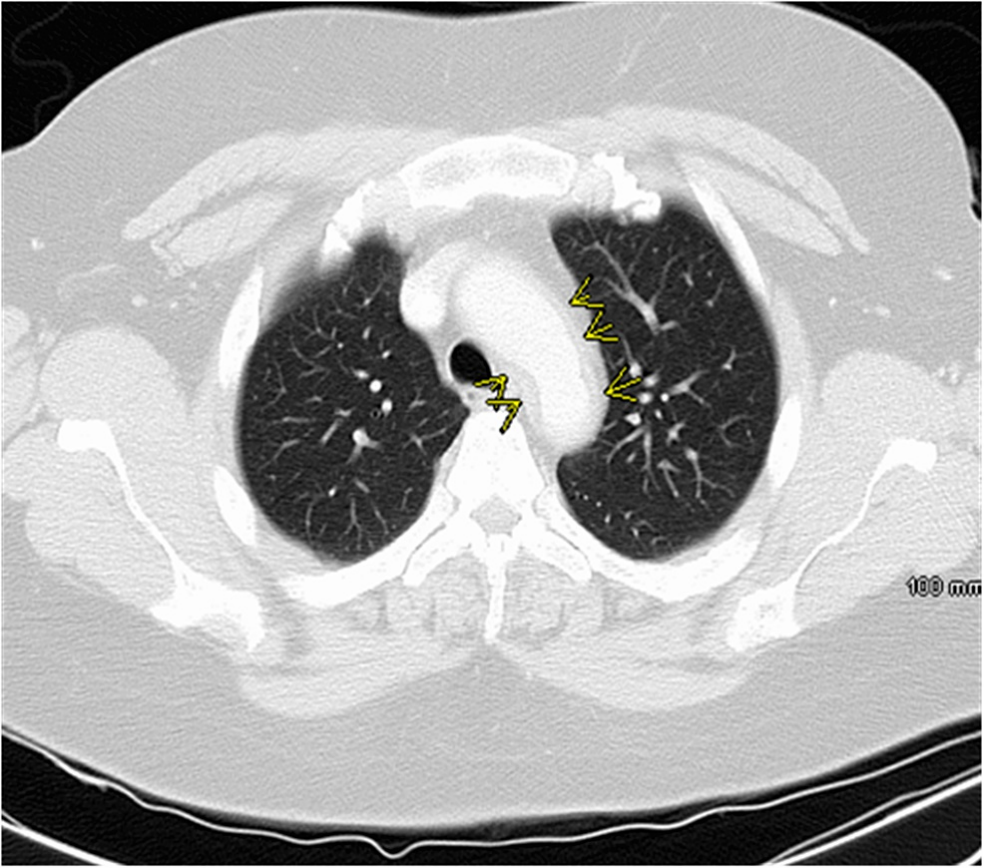
Computed tomography angiography of the chest, axial view The yellow arrow indicates the origin of the mural thickening of the transverse and descending thoracic aorta.

**Figure 4:**
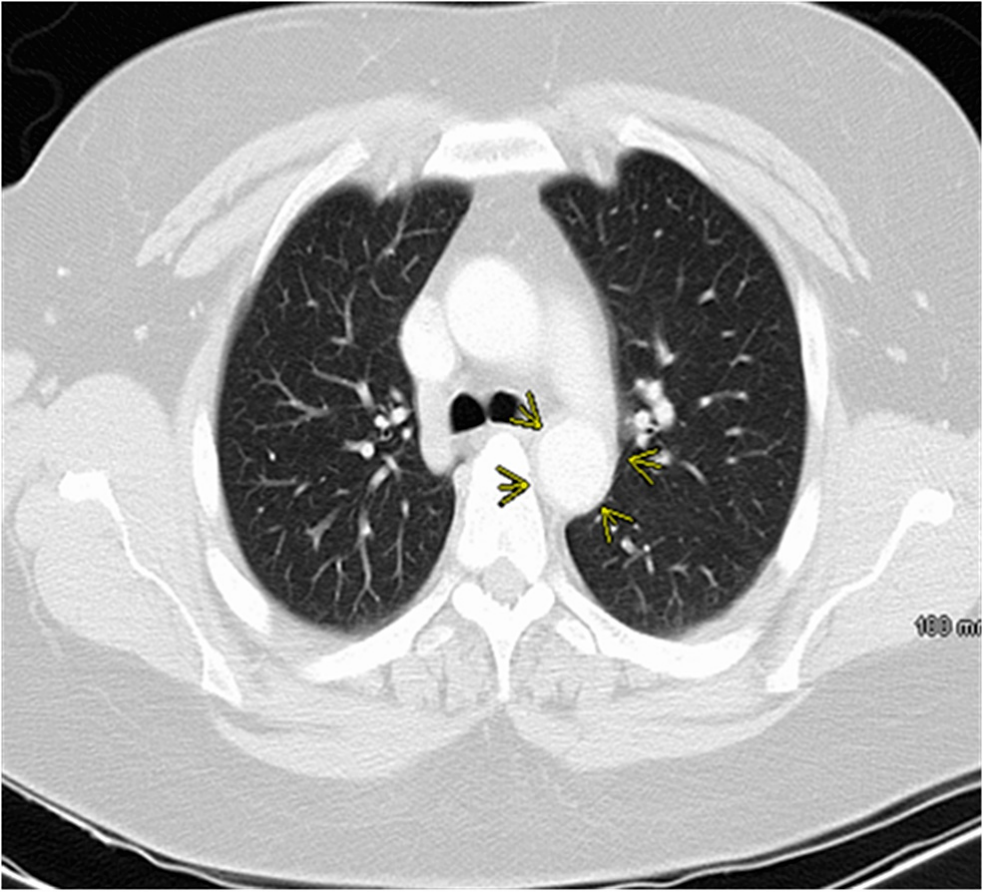
Computed tomography angiography of the chest, axial view The yellow arrow indicates concentric mural thickening of the descending segment of the abdominal aorta.

**Figure 5:**
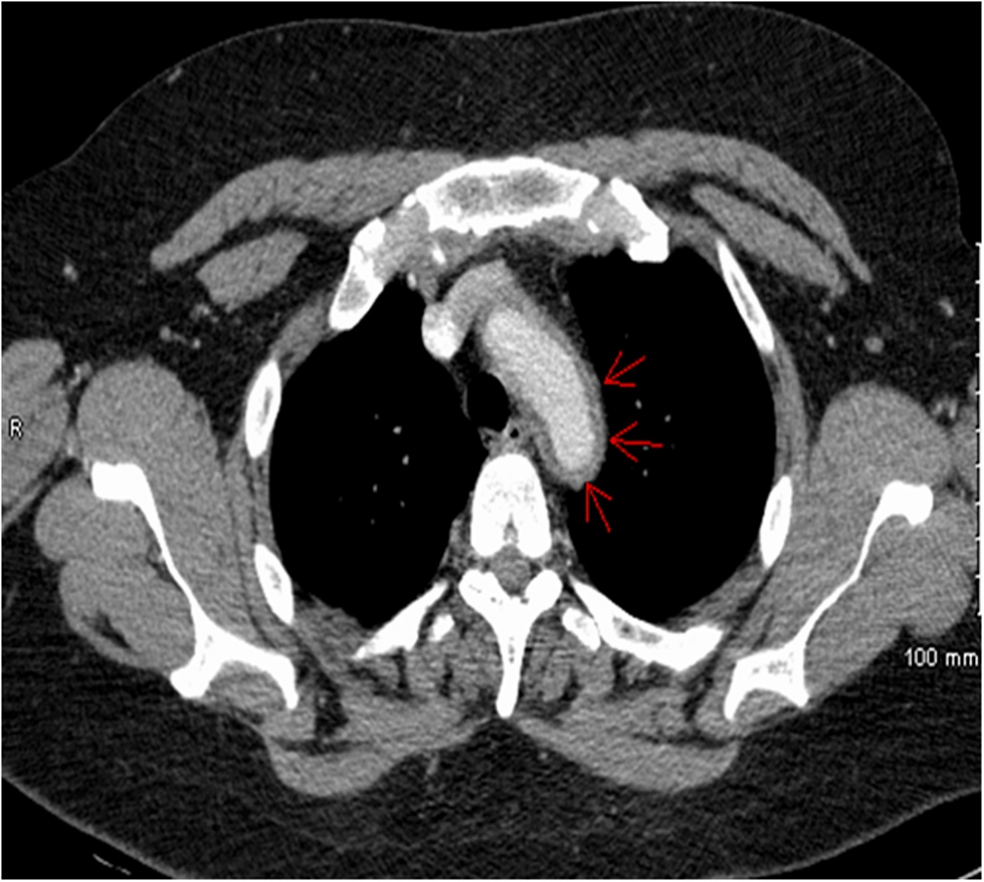
Computed tomography angiography of the chest, axial view The red arrow indicates mural thickening.

Computed tomography angiography of the chest (CTA chest) revealed adventitial/medial thickening of the aorta involving the aortic arch and proximal descending aorta, in keeping with aortitis. No aneurysm or dissection is seen. The proximal left subclavian artery is occluded, which appears to be filled distally via retrograde flow from the left vertebral artery (Figures 2-5).

The vascular surgery team was consulted, and they recommended medical management. She was started on prednisone 1 mg/kg per day for four weeks. The patient improved significantly with the normalization of ESR and CRP. Prednisone was gradually tapered to 20 mg daily for 3 months.

She remains on low-dose prednisone and methotrexate 25 mg daily. She continued to follow-up appointments with vascular surgery and rheumatology.

## Discussion

Takayasu arteritis most commonly affects Asian females, but anyone can be affected [10]. This case describes a less common presentation of TAK in a Caucasian woman that was complicated by subclavian steal syndrome. Most patients tend to present in the “inflammatory phase,” which is characterized by signs of systemic illness such as fever, night sweats, joint pain, and fatigue [9]. If the patient presents with complaints of syncope, as in our case, subclavian steal syndrome should be considered. Common laboratory findings include normocytic anemia and elevation of inflammatory markers such as CRP and ESR. This extensive inflammation can ultimately cause vascular insufficiency due to the intimal narrowing of the affected vessels, causing pulselessness [1,11].

In rare cases, TAK may cause renal artery stenosis leading to hypertension or neurological manifestations if the carotid arteries are affected [12]. Imaging is essential in the diagnosis of TAK. CTA and MRA of the arterial tree will demonstrate smoothly tapered luminal narrowing or occlusion [10,13].  Initial treatment of TAK consists of high-dose steroids for two to four weeks, followed by a timed taper after the patient shows clinical improvement.  It is recommended to use a glucocorticoid-sparing agent in combination with steroids such as methotrexate [10]. In this case, the patient was shown to have the typical signs of TAK: elevated inflammatory markers, abnormal imaging, and coinciding symptoms. In this case, early recognition and treatment of TAK helped prevent further progression and complications of convulsions and strokes. 

The index case is a female of 34 years. In a review, Alnabwani and colleagues accounted for 88.3% of females with an average age of 25 years and a standard deviation of 12.5 years [14]; this further strengthens the fact that incidence is higher in females with ages less than 40 years.  The clinical symptoms and signs of TAK vary and are not specific. Alnabwani and colleagues also state that fever was the most common symptom in 20.93% of cases, followed by chest pain, claudication, and headache in 13.95% of cases. Other less frequent complaints noted are shortness of breath (11.62%), weight loss (9.30%), syncope (6.98%), and night sweats (4.65%). Bradycardia was primarily noted in the radial artery in 15 out of 43 cases reviewed. The onset of lightheadedness, syncope, orthostasis, headaches, strokes, and convulsions are sequelae of subclavian steal syndrome as seen in the index case [4]. Subclavian steal syndrome can also arise from atherosclerotic arterial disease, giant cell arteritis and even intervention for the congenital disorder can result in isolation of the subclavian artery and sacrifice of the proximal subclavian artery in aortic surgery such as Blalock-Taussig procedure done for tetralogy of Fallot, thoracic outlet compression syndrome has also been associated with subclavian artery occlusion, however, this usually involves the subclavian artery distal to the vertebral artery origin [15]. Therefore, a patient required thorough evaluation to ascertain the possible cause and thus determine its management. 

Over a decade ago, Manfrini and colleagues reported similar findings in a middle-aged Caucasian woman, who presented with severe acute pulmonary edema with earlier complaints of weakness, malaise, and fatigue that has protracted for as many as 19 years, she had a 20-year history of hypertension of which her medications were discontinued on account of an episode of syncope with the finding of very low arterial pressure (95/60 mmHg) in the right arm [16]. Further evaluation with angiography, findings of focal narrowing of the abdominal and thoracic aorta and occlusion of both the subclavian arteries and the right coronary artery and severe stenosis of the first marginal obtuse was observed and was diagnosed with TAK. The challenging encounter here was a delay in making the diagnosis of TAK, this is common because there are no specific symptoms for its diagnoses, therefore, a high index of suspicion, the use of clinical criteria, and prompt imaging investigation are required by the clinician to clinch its diagnosis. 

The prevalence of subclavian steal syndrome is said to be between 0.6% to 6%, the actual prevalence is unknown as some patients are asymptomatic, Confirmation of a steal syndrome is usually made by radiologic studies, which include studies that can detect subclavian stenosis and to observe a reversal of flow from the vertebral artery [17]. Ultrasound scan has been used as a screening tool due to accessibility and low cost. It can partly quantify the degree of subclavian stenosis and diagnoses other extra-cranial carotid occlusive disease. more so, the direction of the basilar artery blood flow can be observed with transcranial ultrasound, however, images with higher quality and resolution have been obtained through newer technology modalities, such as CTA and MRA, which are widely used now [13]. 

The mainstay to achieve remission of Takayasu arteritis is the use of glucocorticoids [18]. Recent findings have also shown that conventional disease-modifying anti-rheumatic drugs (cDMARDs) or biologic DMARDs and (bDMARDs) are also used and found to be efficacious in the retardation of angiographic progression [19]. The treatment of choice for symptomatic stenoses is revascularization, either through surgical or endovascular procedures, this is done to relieve critical symptomatic stenoses. The index patient had vascular surgery along with recommended medical management. Studies have shown that the best evidence-based treatments include steroids, to which 50% of patients attain remission, and methotrexate to which a further 50% respond [10].

## Conclusions

Immune-mediated vasculitis such as Takayasu arteritis (TAK) can present with a wide range of symptoms. Subclavian steal syndrome as one of the features has also been described as seen in the index patient. Recurrent syncopal episodes without identifiable cause should raise the index of suspicion of subclavian steal syndrome as the initial presentation of Takayasu arteritis (TAK). Early detection and diagnosis of TAK are essential in the prognosis of TAK. There is a need for further research work to help establish an accurate diagnostic criterion for TAK to help prevent complications due to late presentation.
